# Sequential Two-Photon Delayed Fluorescence Anisotropy for Macromolecular Size Determination

**DOI:** 10.1021/acs.jpcb.3c01236

**Published:** 2023-04-25

**Authors:** Yi-Han Lu, Matthew C. Jenkins, Katherine G. Richardson, Sayan Palui, Md. Shariful Islam, Jagnyaseni Tripathy, M. G. Finn, Robert M. Dickson

**Affiliations:** † School of Chemistry and Biochemistry and Petit Institute of Bioengineering and Biosciences, Georgia Institute of Technology, Atlanta, Georgia 30332-0400, United States; ‡ Department of Physics, School of Applied Sciences, KIIT University, Bhubaneswar 751024, India

## Abstract

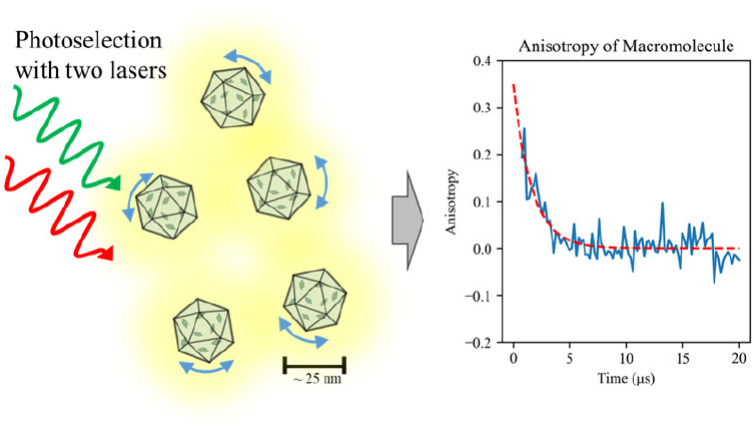

Time-resolved fluorescence anisotropy (FA) uses the fluorophore depolarization rate to report on rotational diffusion, conformation changes, and intermolecular interactions in solution. Although FA is a rapid, sensitive, and nondestructive tool for biomolecular interaction studies, the short (∼ns) fluorescence lifetime of typical dyes largely prevents the application of FA on larger macromolecular species and complexes. By using triplet shelving and recovery of optical excitation, we introduce optically activated delayed fluorescence anisotropy (OADFA) measurements using sequential two-photon excitation, effectively stretching fluorescence anisotropy measurement times from the nanosecond scale to hundreds of microseconds. We demonstrate this scheme for measuring slow depolarization processes of large macromolecular complexes, derive a quantitative rate model, and perform Monte Carlo simulations to describe the depolarization process of OADFA at the molecular level. This setup has great potential to enable future biomacromolecular and colloidal studies.

## Introduction

Hydrodynamic volume is a key molecular property of polymers in solution and can be used as a probe of size, structure, oligomerization state, and interactions with other ligands. Widely used in protein-folding and drug discovery studies,^1−5^ fluorescence anisotropy (FA) measures fluorescence depolarization resulting from rotational motion.^6,7^ Although time-resolved fluorescence anisotropy (FA) can measure hydrodynamic volume in many different contexts, its reliance on rotation during the nanosecond-lived fluorescence lifetime renders it unsuitable to probe dynamics of large proteins, polymers, nanoparticles, and macromolecular complexes.^8^

In FA measurements, excitation is linearly polarized with parallel and perpendicular emission polarizations being collected at a right angle to the excitation beam propagation direction. The fluorescence anisotropy, *r*, is given as^8^
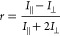
1where *I*_∥_ and *I*_⊥_ are the emission intensities with polarization parallel and perpendicular to excitation polarization, respectively. Continuous-wave (CW) excitation yields a steady-state anisotropy measurement while pulsed excitation produces a time-resolved anisotropy decay. For spherical molecules, the time-resolved FA decay *r*(*t*) can be described as
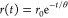
2In this equation, *t* is time, *r*_0_ is the fundamental anisotropy and θ is the rotational correlation time. Fundamental anisotropy is a factor related to the angle between the absorption and emission transition. Rotational correlation time is proportional to the hydrodynamic volume of the molecule (*V*) and is given by^9^
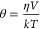
3in which η is the viscosity of solution, *k* is Boltzmann’s constant, and *T* is the temperature. While measurement of steady-state anisotropy provides a simple way to detect molecular interaction and conformational change, molecular volume can only be obtained when other parameters are known.^8^ Therefore, time-resolved anisotropy is a more convenient method for molecular volume measurement.

Although it has several advantages, including being nondestructive, rapid, and highly sensitive, the major limitation of FA measurements is that it is only suitable for measuring small molecules. For large biomolecules, the rotational correlation time θ can easily exceed 100 ns.^8,10,11^ Because the fluorescence lifetime of most commonly used probes is less than 5 ns,^12^ very few photons are detectable at the long delays needed to measure long rotational correlation times, θ, resulting in very poor sensitivity and precluding most such measurements of complexes above 10 nm in diameter. Potential solutions to this problem include fluorophores with extended emissive lifetimes or to utilize long-lived phosphorescence in anisotropy measurements.^13,14^ Although specialized fluorophores with lifetimes of up to ∼20 ns have been reported,^15^ the rotational lifetime scaling with the third power of molecular radius only gives a modest increase in size range using FA. Triplet-state transient absorption has been used for long-term anisotropy,^16^ but such transient absorptions require high concentrations and suffer from low sensitivity overall. Strong emission is crucial for good anisotropy measurements, but the typically low quantum yield of phosphorescence increases the difficulty of measuring phosphorescence anisotropy.

**Figure 1:**
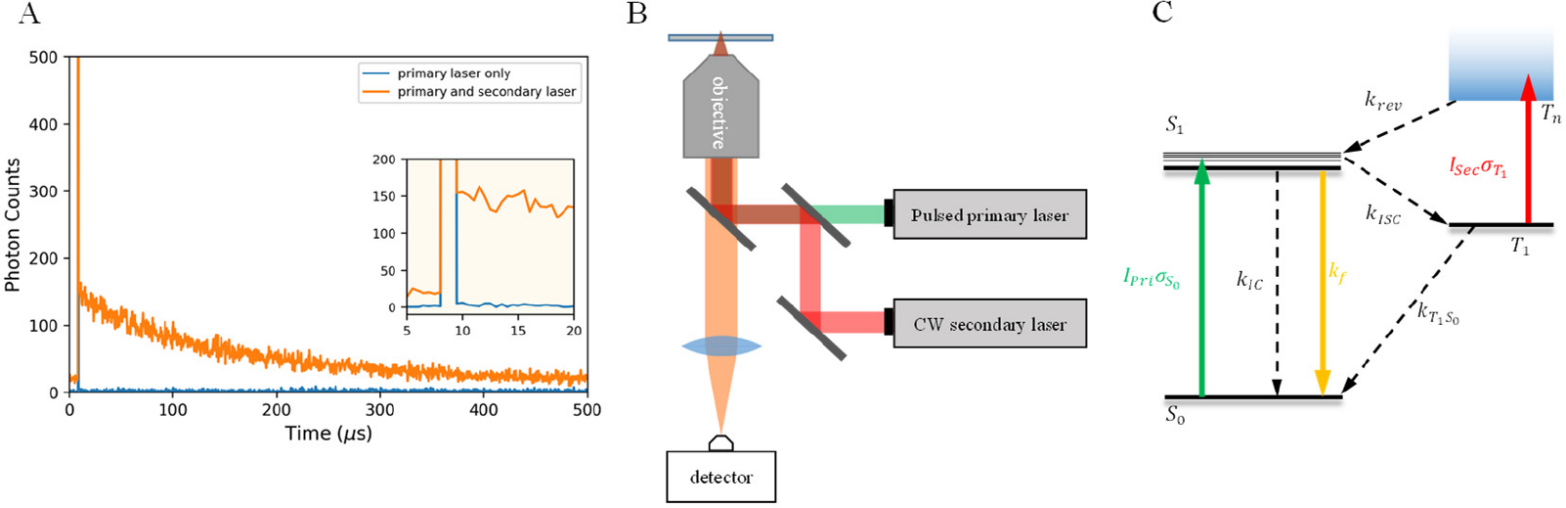
(A) OADF time trace of rose bengal immobilized in poly(vinyl alcohol). The blue curve is the prompt fluorescence time trace from pulsed primary laser excitation alone. The orange curve is fluorescence under both pulsed primary and CW secondary laser excitation. The delayed fluorescence decays slowly with a 137 μs lifetime. The inset plot shows a zoomed-in view of primary-only decay. (B) Diagram of the OADF microscope. A pulsed primary laser is used for exciting the fluorescent dye. A red-shifted continuous secondary laser coilluminates the sample. (C) Jablonski diagram showing the path of primary laser-induced prompt fluorescence (*I*_*pri*_) and secondary laser-induced OADF (*I*_*sec*_). The secondary laser continuously repumps the triplet-state population back to the emissive S_1_ state with a rate much slower than that of fluorescence decay, causing long-lived OADF.

To solve this problem, we utilize triplet shelving and photoinduced reverse intersystem crossing to extend fluorescence anisotropy well into the microsecond regime. This sequential two-photon optically activated delayed fluorescence (OADF)^17−19^ has been demonstrated in cyanine dyes, xanthene dyes, fluorescent proteins, and DNA-wrapped silver nanoclusters to extend ns-lived fluorescence to many hundreds of microseconds beyond the initial pulsed laser excitation while maintaining polarization (Figure 1).^17−20^ A pulsed primary laser excites the ground-state (S_0_) to an excited singlet state (S_1_). While most of the S_1_ population goes back to the S_0_ state within a few nanoseconds producing prompt fluorescence, a fraction of the S_1_ population intersystem crosses to the long-lived dark state (D_1_). A red-shifted secondary laser excites the T_1_–T_*n*_ transition after some delay, causing some reverse intersystem crossing (RISC) to regenerate the S_1_ state at a later time, followed by ns emission to regenerate the S_0_ state (Figure 1C).

The long-lived triplet state prevents excited-state photon emission over short time periods. However, unlike phosphorescence, ns emission can be triggered by long wavelength secondary excitation delayed by many microseconds from the initial excitation pulse. This sequential two-photon process results in background-free ns-lived fluorescence, up to several hundred microseconds after the initial pulsed primary excitation, maintaining the polarization selection from the initial primary excitation pulse.^20^

In this study, we utilize OADF to extend the range of time-resolved FA such that long rotational correlation times of large macromolecules can be directly measured. Using photophysical rate equations, we derive a model for optically activated delayed fluorescence anisotropy (OADFA) and validate the model with Monte Carlo simulations to describe the depolarization process of OADFA at the molecular level. These simulation results were used to guide the construction of a time-resolved optically activated delayed fluorescence anisotropy (OADFA) microscope to probe rotational correlation times of nanoparticles and large biomolecules. The novel methodologies described herein studies have opened a new window into analyzing the solution phase properties of biopolymers and nanoparticles with diameters even exceeding 50 nm.

## Experimental Methods

### Rose Bengal Silica Nanoparticle (RbSNP) Synthesis

The RbSNP synthetic procedure consists of two parts: synthesis of the rose bengal (Rb) precursor and synthesis of the silica particle. To synthesize the precursor, 0.1 mmol of Rb and 0.2 of mmol *N*-ethoxycarbonyl-2-ethoxy-1,2-dihydroquinoline (EEDQ) (both from Sigma-Aldrich) were dissolved in 1 mL of methanol. After stirring in the dark at room temperature for 30 min, 0.15 mmol of (3-aminopropyl)triethoxysilane (APTES) was added to the mixture. The mixture was then incubated in the dark at room temperature with constant stirring for 24 h.

To synthesize the silica nanoparticles, 640 μL of polyoxyethylene nonylphenylether (IGEPAL CO-520, Sigma-Aldrich) was added into 9.2 mL of cyclohexane, and the mixture was sonicated for 30 min. Rb-precursor was then added into the mixture and incubated in the dark at room temperature for 30 min. The volume of Rb-precursor added depends on the desired particle size (Figure S1). After incubation, 110 μL of tetraethoxysilane (TEOS) was added and the mixture was alkalized with 164 μL of 28% aqueous NH_4_OH. After stirring the mixture overnight followed by 30 min sonication, nanoparticles were collected by centrifugation and purified with several washing cycles. In each washing cycle, sample was centrifuged at 7200 rpm for 15 min. The supernatant was removed, and the precipitate was resuspended in water. Nanoparticle sizes were characterized by transmission electron microscopy (JEOL 100CX-II TEM). To prepare the TEM sample, RbSNP was suspended in diH_2_O and pipetted onto the carbon surface of 200 mesh holey carbon–copper TEM grids (Electron Microscopy Sciences). Grids were allowed to air-dry for 60 min prior to imaging.

### mVenus Expression and Purification

The mVenus expression followed the procedure published in previous work.^21^ N-terminal His_6_-tag mVenus plasmid (gifted by Prof. Fahrni) was transformed into chemically competent *E. coli* BL21(DE3) cells via heat shock. Single colonies were selected and cells were grown in the presence of kanamycin at 37 °C with shaking at 250 rpm to an optical density at 600 nm (OD_600_) of 0.6. Protein expression was induced by IPTG and the resulting culture was pelleted. The cell pellet was lysed via sonication and the lysate was run through a 1 mL HisTrap FF Nickel affinity column (GE Healthcare) on an ÄKTAprime chromatography system. Eluted fractions were buffer exchanged (PBS; pH 7.4), concentrated via a 10 kDa MW cutoff filter, and purity was confirmed by SDS-PAGE. Protein concentration (2.7 mg/mL) was also quantified using a Bradford protein assay with BSA as a standard.

### mV-VLP Expression and Purification

The mVenus gene was cloned into a bicistronic plasmid, pKMJ2, containing the Qβ coat protein gene and an encoded N-terminal Rev-peptide for the fluorescent protein using the plasmid’s EagI and XhoI restriction sites.^22,23^ Each gene (Qβ CP and Rev-mVenus) had its own ribosomal binding site for translation of each gene separately from the same transcribed mRNA strand. Successful gene incorporation was verified by Sanger sequencing.

The pKMJ2 plasmid was transformed into chemically competent *E. coli* BL21(DE3) cells (New England Biolabs) and grown on 2YT agar plates containing 50 μg/mL streptomycin. For culture growth, a single colony was inoculated into 25 mL of 2YT medium supplemented with streptomycin and grown to saturation overnight at 37 °C in a shaking incubator. The next morning, cells from the saturated starter culture were seeded into 500 mL of fresh 2YT media supplemented with streptomycin and were grown at 37 °C in a shaking incubator until the OD_600_ value reached 1.0. Protein expression was induced via the addition of a final concentration of 1 mM IPTG and was carried out at 26 °C for 20 h prior to harvesting the cells via centrifugation in a JA-16.250 rotor at 8,000 rpm for 10 min at 4 °C. Cell pellets were stored at −80 °C until purification.

Cells from the entire 500 mL expression culture were resuspended in 50 mL of 0.1 M potassium phosphate buffer (pH 7.5) and were lysed by sonication (5 s sonication pulses at 50 W followed by 5 s of rest for 10 min of total sonication time). The lysate was clarified in a JA-17 rotor at 14,000 rpm for 15 min at 4 °C. VLPs were salted out of the clarified lysate by adding a final concentration of 0.265 g/mL (NH_4_)_2_SO_4_ and incubating the lysate at 4 °C for 1 h on a rotisserie. The precipitated VLPs were collected by centrifugation at 14,000 rpm for 15 min at 4 °C in a JA-17 rotor, and the resulting supernatant was decanted. The solid pellets were resuspended in 8 mL of fresh 0.1 M potassium phosphate buffer (pH 7.5) and hydrophobic contaminants were removed by performing an organic extraction with an equal volume of a 1:1 *n*-butanol/chloroform organic mixture. The VLP-containing aqueous layer was resolved by centrifugation at 12,000 rpm for 12 min at 4 °C in a JA-17 rotor, and then the VLPs were further purified in 10–40% sucrose density gradients prepared in phosphate buffer (SW32 rotor, 28,000 rpm for 4 h at 4 °C). The VLPs were aspirated out of the sucrose gradients and were concentrated by centrifugation in a Type70-Ti rotor at 68,000 rpm for 2 h at 4 °C. The final VLP pellet was resuspended in fresh phosphate buffer and passed through a 0.2 μm PES syringe filter. Purified VLPs were stored at 4 °C for immediate use or were frozen at −80 °C for long-term storage. The final protein concentration was determined using a Coomassie Plus Bradford Assay Kit (Pierce) with bovine serum albumin as the protein standard.

For DLS assessment, VLP samples were diluted to 0.1–0.125 mg/mL in 0.1 M potassium phosphate buffer (pH 7.5). DLS measurements were recorded using a Dynapro DLS instrument (Wyatt Technologies) with an instrument laser power between 10 and 20%, an attenuation value between 10 and 30% and a laser wavelength of 830 nm.

For TEM imaging, 8 μL of VLP sample [0.05–0.1 mg/mL in 0.1 M potassium phosphate buffer (pH 7.5)] were pipetted onto the carbon surface of 300 mesh carbon–copper Lacey Formvar TEM grids (Ted Pella Inc.) for 90 s before blotting the grids dry on filter paper. The grids were washed by floating them successively on two 0.5 mL drops of double-distilled H_2_O (10 s incubation per drop) before blotting the grids dry again on filter paper. Negative staining was then performed by pipetting 8 μL of 2% (w/v) uranyl acetate onto the grid surface for 60 s before blotting the grid on filter paper. Grids were allowed to air-dry for a minimum of 5 min prior to imaging. TEM images were collected on a Hitachi HT7700 microscope operating at an accelerating voltage of 120 kV.

### Normal FA and OADFA Measurement

**Figure 2:**
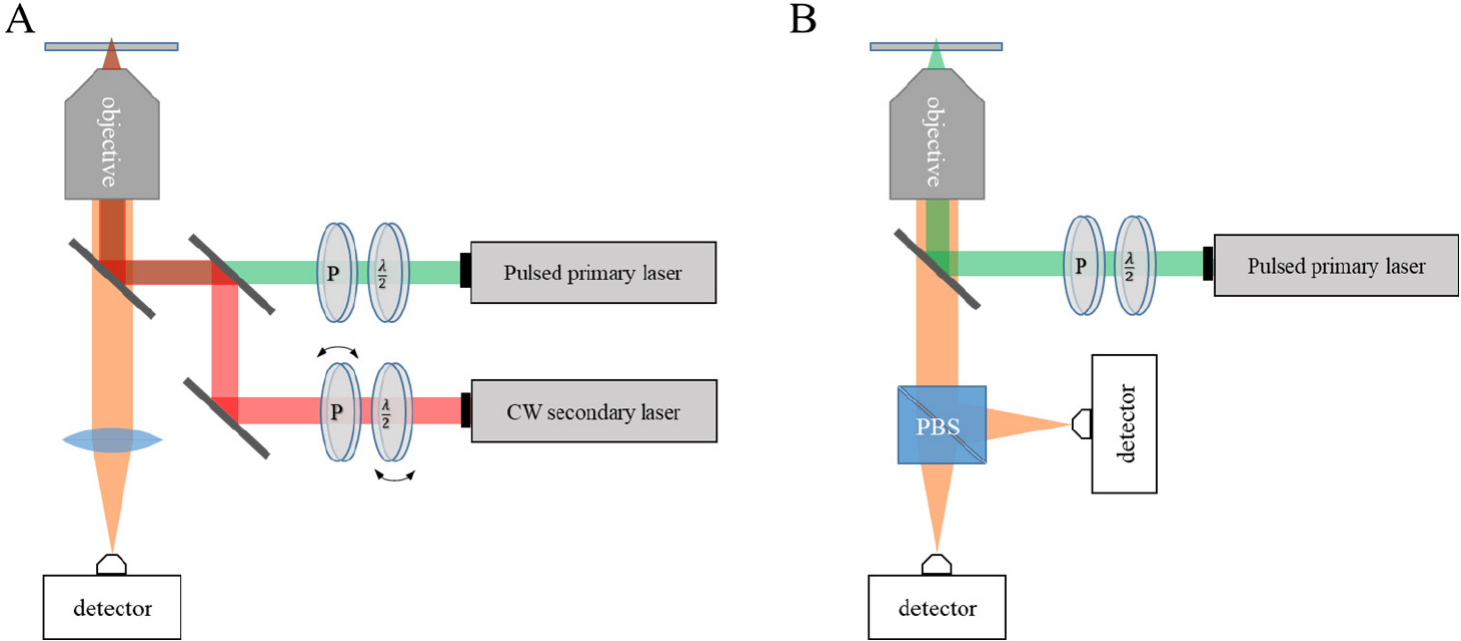
(A) OADFA setup. Sample on the slide is coilluminated by two lasers to obtain OADF. The polarization of each laser is controlled by a half-wave plate (λ/2) and polarizer (P). In the experiment, we fixed the primary laser polarization and adjusted the secondary polarization to be parallel or perpendicular to primary polarization. (B) Normal FA setup. Only one laser is used for excitation, and the polarization is controlled as in (A). The emission signal passes through a polarizing beam splitter (PBS) and the resulting *I*_∥_ and *I*_⊥_ are collected by two detectors, respectively.

Both FA microscopies are performed on an inverted microscope (Olympus IX70 or IX71) with a 60X water objective (Olympus UPlanAPO 60*x*/1.20 NA). All experiments were in a confocal arrangement with a 100 μm multimode fiber (Thorlabs) as the pinhole and directing the fluorescence signal to a photon-counting avalanche photodiode (APD, PerkinElmer or Excelitas). Photon counts are collected by a PCI-6602 Counter (National Instruments). For the experiments with time bin smaller than 500 ns, a Becker & Hickl SPC-130EM single photon counting module was used for recording photons in FIFO mode. A 532 nm pulsed diode laser (Picoquant LDH, ∼100 ps pulse width) served as the primary laser. A CW 830 nm laser (Picoquant LDH) was used as the secondary laser for coillumination to generate the OADF signal. Polarization of each laser excitation was independently controlled (Figure 2). The laser beams were combined prior to entering the microscope with a dichroic mirror. A CCD (Andor Technology, iXon DV885) was used to confirm two beams were spatially overlapped and focused on the sample. A band-pass filter was placed in the emission light path to block primary and secondary excitation and collect fluorescence.

## Results and Discussion

### Time-Resolved OADFA Setup and Theory

To combine OADF and FA microscopies, both primary and secondary polarizations are independently controlled (Figure 2). Unlike the normal fluorescence anisotropy microscopy (Figure 2B) which needs an emission analyzer (polarizer or polarizing beam splitter) to measure *I*_∥_ and *I*_⊥_ in eq 1, there is no analyzer in the time-resolved OADFA setup. As sequential two-photon OADF results from secondary excitation, the secondary laser polarization acts as the analyzer in OADFA and allows all fluorescence to be collected on a single detector. Importantly, the beam diameters for primary and secondary excitations relative to the objective back aperture diameter determine the polarization purity as small diameters limit excitation numerical aperture (NA). The full 1.2 NA of the water immersion objective is still used in collection, but since polarizers are not used in OADFA collection, polarization mixing due to high NA objectives is greatly reduced when the back aperture is only partially filled. We used beam diameters of ∼3 mm relative to the 8 mm back aperture diameter, giving polarization purity of ∼340 for in vs out of plane polarization components and ∼8000 for X vs Y in plane components in this sequential two photon setup (product of primary and secondary intensities). This is a significant advantage for high NA collection on a microscope as normal polarizer-based fluorescence anisotropy would be much more strongly affected by any polarization mixing due to high NA collection.^24,25^

Primary excitation photoselects emitters in OADFA as in normal FA, with the probability of a molecule being excited being proportional to |μ⃗_*abs*_·*E⃗*_*ex*_|^2^, where μ⃗_*abs*_ is the absorption dipole moment of molecule and *E⃗*_*ex*_ is the electric field of the primary excitation.^26^ Thus, the excitation probability *P*(*ex*) is proportional to *I*_*laser*_ cos^2^ α, where *I*_*laser*_ is the intensity of the illuminating laser and α is the angle between the absorption dipole moment and the laser polarization. Therefore, the effective excitation intensity experienced by a given molecule can be represented as *I*_*laser*_ cos^2^ α. Integrating over all orientations would give the average excitation for all molecules in solution. This photoselection gives a bulk cos^2^ α dependence on molecular orientation, α, relative to excitation polarization. Assuming parallel absorption and emission transition dipoles, emission intensity is then also polarized, with analyzers in the emission path giving additional cos^2^ α and sin^2^ α factors for collected parallel and perpendicular polarizations, respectively.

In our FA microscopy experiments, we fix the illuminating polarization and split emission into *I*_∥_ and *I*_⊥_ with a polarizing beam splitter (Figure 2B). The excitation and emission intensities can be defined as
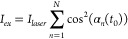
4
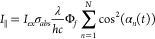
5
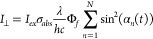
6in which *I*_*laser*_ is the intensity of the pulsed excitation laser, σ_*abs*_ is the absorption cross section, and Φ_*f*_ is the fluorescence quantum yield. Due to rotation, the angle between molecular orientation and laser polarization, α_*n*_, is a function of time. α_*n*_(*t*_0_) indicates the angle for molecule *n* at *t* = 0, which determines the excitation rate when a pulsed laser is used. The molecular orientation affects not only the excitation rate but also the emission from each molecule. Therefore, the overall excitation (*I*_*ex*_) and emission intensities (*I*_∥_, *I*_⊥_) should be the sum of the contribution from each molecule in a system of *N* molecules (eqs 4–6).

Fluorescence anisotropy actually measures fluorescence polarization intensity differences resulting from orientation changes (α_*n*_(*t*) to α_*n*_(*t*′)) typically on the ns-scale occurring between molecular excitation and when the molecule emits. In OADFA, we use the secondary laser as the analyzer, giving expressions for *I*_*ex*_, *I*_∥_ and *I*_∥_ as
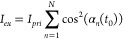
7
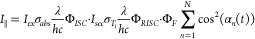
8
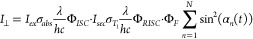
9*I*_*pri*_ and *I*_*sec*_ are the laser intensity of primary and secondary laser, respectively. Φ_*ISC*_ and Φ_*RISC*_ are the quantum yields of intersystem crossing and reverse intersystem crossing, respectively. Equations 8 and 9 describe how molecules enter their triplet state and go through the photoselection process with polarized secondary excitation. Comparing eqs 4–6 to eqs 7–9, OADFA is very similar to normal FA measurement, just extended to the long OADF time scales to provide a very wide window for measurement of slow rotation. Although no emission polarizers are used in OADFA, the primary and secondary laser photoselection processes give overall cos^4^(α) and cos^2^(α) sin^2^(α) orientation dependences for parallel and perpendicular dual laser excitation, respectively.

### Monte Carlo Simulation

As with our rate-based simulations of OADF,^17,18,20^ coupled photophysical rate equations (eq 10) are readily integrated under pulsed primary and continuous-wave (cw) secondary polarized excitations to yield state populations at any time point.
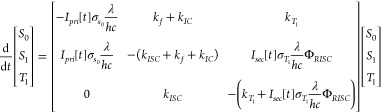
10

**Figure 3:**
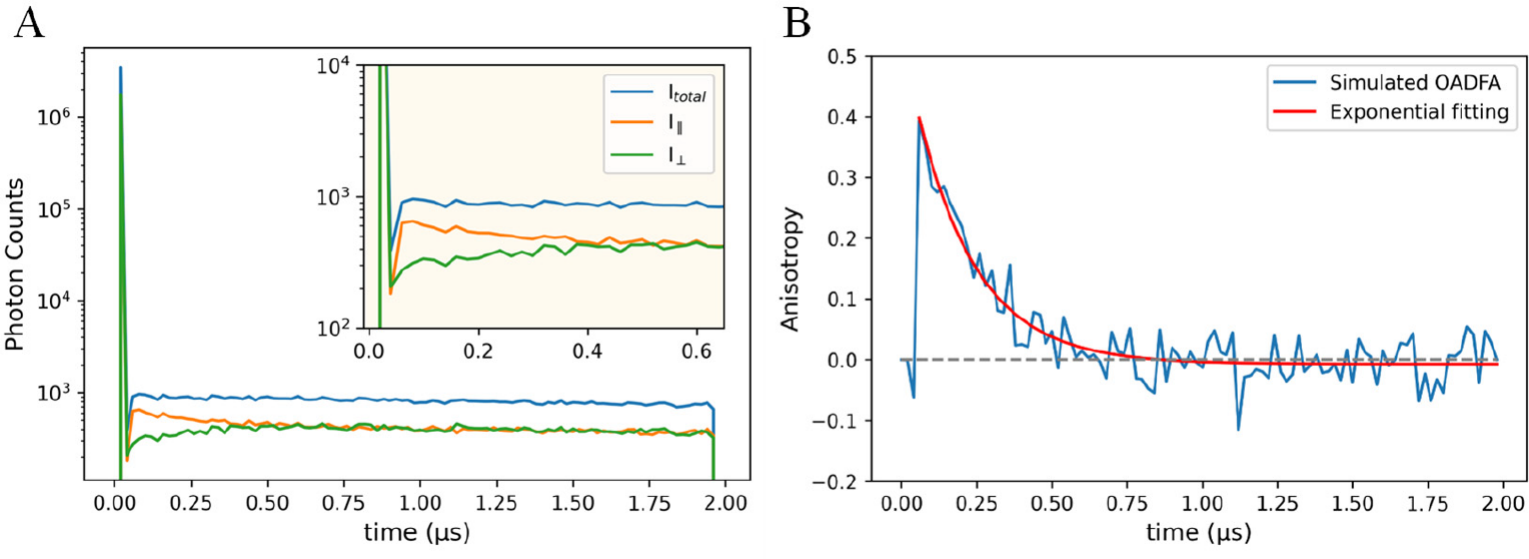
(A) Monte Carlo-calculated time traces of *I*_∥_, *I*_⊥_ and total emission intensity of OADF (*I*_*total*_). The *I*_*total*_ in the OADFA experiment is defined as *I*_∥_ + *I*_⊥_. In this simulation, the rotational correlation time (θ) is set to 200 ns. (B) Simulated OADFA time trace (blue curve) calculated from *I*_∥_ and *I*_⊥_ in plot A. Equation 2 is used for fitting (red), which gives *r*_0_ = 0.41 and θ = 199.5 ns.

The singlet absorption cross section (σ_*s*_0__), triplet absorption cross section (σ_*T*_1__) and reverse intersystem crossing quantum yield (Φ_*RISC*_) of rose bengal were reported in previous studies.^20,27^ However, a new model is required for OADFA because molecular orientation changes between primary and secondary excitation events affect excitation rates, making the rate “constants” time and orientation dependent, preventing the integration of eq 10 to determine state populations and measured anisotropies. Therefore, we performed Monte Carlo simulations of time dependent orientations and photophysical populations to understand and model OADFA. For each molecule (of typically 5 × 10^6^ per simulation), we generated a rotational time trace with a particular rotational diffusion coefficient governed by a Wiener process.^28,29^ Combining eqs 7–10, the excitation rate for molecules in the ground state is given by . Similarly, the excitation rates for molecules in the triplet state are  and  when secondary polarization is parallel and perpendicular to primary polarization, respectively. Both excitation and emission events were simulated as random processes sampled from distributions corresponding to the average (orientation-dependent) excitation and emission rates. Summing millions of individual molecule photon counts and excitation cycles for each time bin produced simulated time traces of *I*_∥_ and *I*_⊥_ (Figure 3A). For OADFA, we define anisotropy as
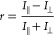
11This anisotropy is slightly different from the definition of normal fluorescence anisotropy (eq 1). As no emission analyzer is used in OADFA, the OADFA total emission intensity, *I*_*total*_, is equal to *I*_∥_ + *I*_⊥_, as is obtained from eqs 8 and 9. The OADFA time trace is calculated from eq 11 at each time delay and fitted to eq 2 to obtain the rotational correlation time. Beyond the prompt fluorescence region, both *I*_∥_ and *I*_⊥_ decay due to triplet-state decay, but *I*_∥_ also exhibits rotational diffusion decay while *I*_⊥_ increases in time as rotation improves overlap with perpendicular secondary excitation at longer times. Similar increasing and decreasing decays also occur in normal FA when rotational correlation times are comparable to or longer than the ∼ns fluorescence lifetimes.^30^ The normalized difference of OADF *I*_∥_ and *I*_⊥_ curves (eq 11) then yields the OADFA (Figure 3A) and accurate sizes from fitting the long-time rotational anisotropy (Figure 3B).

While there are significant advantages to OADFA vs ordinary FA using high NA collection, and only OADFA enables slow rotation to be measured, the OADFA time trace has a short “blind time” during the first few nanoseconds after the primary laser pulse (Figure 3B), which is not present in the normal FA measurement. This phenomenon results from the strong prompt fluorescence signals that dominate over OADF within the first two collection bins, so the prompt fluorescence signal of *I*_∥_ will always be equal to that of *I*_⊥_ in the no analyzer experimental configuration of OADFA (Figure 3A). One should thus optimize bin width so it is wide enough to collect sufficient photons/bin for good signal/noise while minimizing the two-bin blind time. The second difference is that the fundamental anisotropy (*r*_0_) defined in eq 2 represents a different physical property in OADFA. In the normal FA experiment, *r*_0_ is related to the angle between the absorption and emission dipoles.^8^ In OADFA, on the other hand, *r*_0_ represents the angle between the singlet and triplet absorption dipole.

### OADFA Measurement of Rose Bengal Silica Nanoparticles

**Figure 4:**
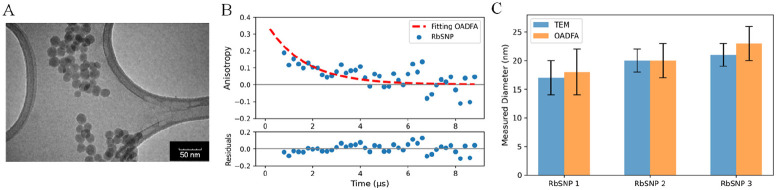
(A) TEM image of RbSNP. (B) OADFA measurement of RbSNP. The blue scatter is the OADFA time trace, and the red curve is the fitting result. The rotational correlation time (θ) from the fitting is 1.5 ± 0.6 μs, and the particle diameter calculated from θ is 23 ± 3 nm. (C) Comparing the particle diameters measured by TEM (blue) versus OADFA (orange). Three RbSNP samples with different sizes were tested. The diameters from TEM are 17 ± 3, 20 ± 2, and 21 ± 2 nm for RbSNP samples 1, 2, and 3. The OADFA results for RbSNP1, -2, and -3 are 18 ± 4, 20 ± 2, and 23 ± 3 nm, respectively.

The cubic dependence of rotational correlation time on hydrodynamic radius effectively limits fluorescence anisotropy to small to medium sized proteins. At 66 kDa molecular weight, serum albumins already exhibit rotational correlation times of 41.7 ns at 25 °C—a time that is much longer than the typical 3–5 ns fluorescence lifetime of most dyes.^31^ To extend this range, we tested OADFA on much larger nanoparticle and protein complexes and compared the OADFA-determined sizes with other size-measuring approaches. As rose bengal (Rb) exhibits strong OADF,^20^ we synthesized a series of rose bengal-embedded silica nanoparticles (SNPs),^32,33^ controlling nanoparticle size by adjusting the Rb to tetraethoxysilane ratio (Figure S1). Particle sizes for each batch were determined both by TEM and by OADFA and compared (Figure 4). The OADFA data is fitted with eq 2 after removing the first few time points to ensure that the fitting is not affected by the prompt fluorescence (Figure 4B). Equation 3 and the measured θ enabled particle diameters to be determined (using *T* = 293 K and solution viscosity of 1.0005 cP). For particles with average diameters ranging from 15 to 24 nm, OADFA-determined particle sizes (Figure 4C) match the TEM-determined sizes quite well.

### OADFA Measurement of Virus-like Particle

**Figure 5:**
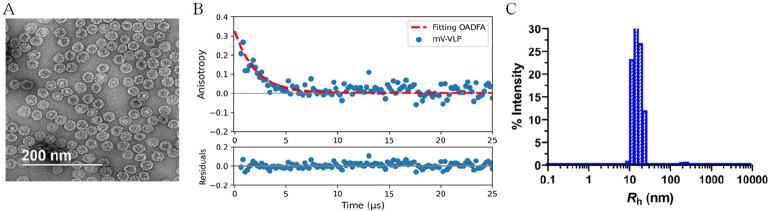
(A) TEM image of mV-VLP. (B) OADFA time trace of mV-VLP. The hydrodynamic radius from this data is 12.5 ± 0.6 nm, compared to 14 ± 1 nm reported by dynamic light scattering (panel C).

For better control of particle size and more biological relevance, we used OADFA to determine the size of Qβ, a recombinant virus-like particle (VLP) derived from an *E. coli* bacteriophage *(Leviviridae)*.^34,35^ This self-assembled macromolecular assembly forms a highly stable container^34−36^ that can be used for cargo delivery,^27,30^ and VLPs were loaded with the fluorescent protein mVenus—a fluorescent protein we have shown to exhibit OADF.^21,36,37^ Qβ-mVenus OADFA yields a measured radius of 12.5 ± 0.6 nm, which is very close to the radius measured by dynamic light scattering (DLS) (Figure 5C) and TEM (Figure S3).

### Viscosity Effect on Rotational Correlation Time

The rotational correlation time is a macroscopic property originating from rotational Brownian motion.^8,13,38^ Therefore, θ is affected not only by particle size but also by solution viscosity (η) and temperature (*T*). The relationship between these factors is indicated by eq 3. mVenus yields strong OADF, but the weaker OADF is dominated by prompt fluorescence at short delays from primary excitation. By adding sucrose into solution, the viscosity was increased without quenching the fluorescence emitted from mVenus. We measured the θ of mVenus in sucrose-free PBS buffer (η = 1.0005 cP at 293 K) by normal FA and 60% (w/w) sucrose–PBS buffer (η = 56.76 cP at 293 K) by OADFA separately to compare the experimental value and theoretical value.^39,40^ The result suggests that θ of mVenus has increased from 16.8 ns (Figure S2A) to 860 ns (Figure S2B) after the sucrose was added. This OADFA result is very close to the theoretical θ (810 ns) for a 26.9 kDa mVenus molecule based on our calculation.^41,42^

### The Range of Detectable θ in OADFA Measurement

**Figure 6:**
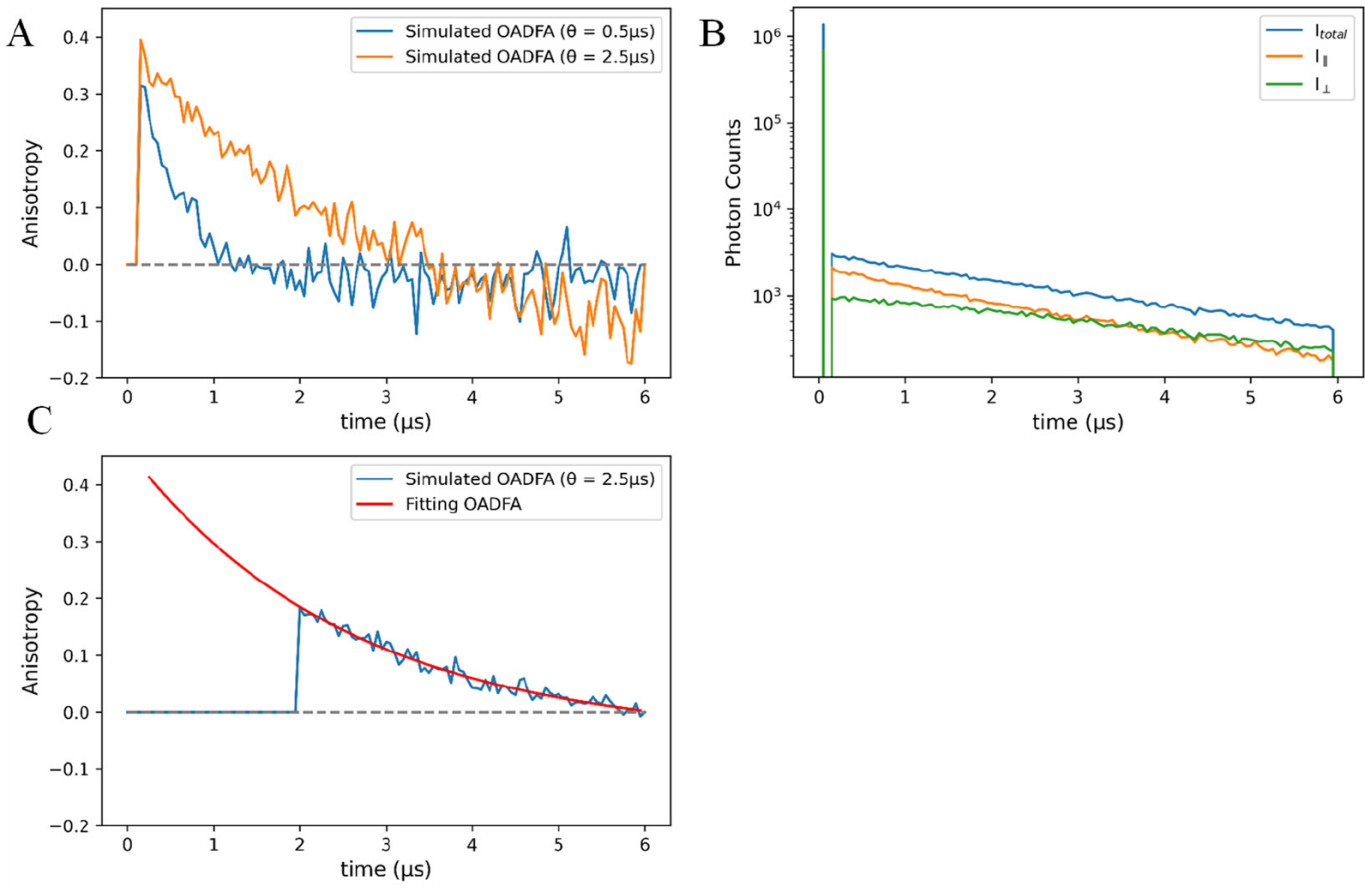
(A) Simulated OADFA with θ set as 0.50 μs (blue) and 2.50 μs (orange). When θ = 0.50 μs, the time trace decays slightly below 0 but still within the range of error and does not affect the fitted result (fitted θ = 0.51 μs). Nevertheless, when θ is set to be 2.50 μs, the anisotropy value could decay far below zero. This makes the fitted result unreliable (fitted θ = 3.32 μs). (B) Simulated OADF intensities time trace with θ set as 2.50 μs. *I*_∥_ decays faster than *I*_⊥_ and eventually become smaller than *I*_∥_ in the tail region (3.5 to 6 μs). (C) Simulated delayed-secondary OADFA time trace. The secondary laser is turned on 2 μs after the primary pulse. The anisotropy value stays above zero in the tail region. Even though the initial part of the time trace is missing, fitting with only the tail part still gives a more reliable result (fitted θ = 2.55 μs) compared to the result from Figure 6A.

OADFA is a promising new approach for macromolecular and nanoparticle size determination in a size range that is inaccessible by fluorescence anisotropy. We have utilized OADFA to extend measurable size ranges to ∼30 nm diameters, which show ∼1 μs rotational correlation decays. To probe the limits for size measurement, Monte Carlo simulations were run on larger complexes, but for such slowly rotating species, anisotropy time traces deviate from eq 2 at long times (Figure 6A), with *I*_∥_ decaying much faster than *I*_⊥_. Resulting from photoselection-induced triplet-state population being depopulated faster when secondary polarization is parallel to primary polarization than when it is perpendicular to primary polarization, apparent negative anisotropies at long delays are seen (Figure 6B). For a molecule emitting delayed fluorescence in OADFA, photoselection of slowly rotating species by the primary laser results in higher triplet excitation rates for secondary laser parallel to primary excitation than for it being perpendicular to primary excitation. This photoselection causes the secondary laser-induced decay to be higher for *I*_∥_ than for *I*_⊥_. Therefore, when primary and secondary polarization are parallel to each other, most of the molecules in the triplet state are also aligned to the secondary laser and have higher chance to be excited, depopulating the triplet state faster. In the fast-rotating scenario, the molecular orientation will quickly become isotropic, making the depopulation process become less sensitive to the photoselection-dependent secondary excitation rate differences. For large complexes, one can mitigate this effect and extend the measurable range of particle size in OADFA by introducing a delay before turning the secondary laser on (Figure 6C). After the primary pulse, the molecules in the triplet state experience a short period (1–2 μs) to rotate without being depopulated the by secondary laser. When the secondary laser is turned on to generate OADF, slowly rotating complexes still exhibit anisotropy with the same correlation decay, but one can probe the later portion of the decay curve without having differentially depopulated the triplet state at short decay times. Introducing this delayed secondary excitation mitigates any effects of photoselection differentially depopulating the triplet-state depopulation with parallel and perpendicular secondary excitations, while not affecting the measured rotational correlation times.

## Conclusions

Although measuring molecular volume by time-resolved fluorescence anisotropy is fast and simple, the detectable range is limited by typically short fluorescence lifetimes. In general, the molecules that can be measured accurately are smaller than 25 kDa. Triplet shelving, followed by optically induced reverse intersystem crossing enables OADFA to avoid the fluorescence lifetime limitation.

Guided by Monte Carlo simulations, we illustrate the OADFA process of molecules in different sizes and calculate their rotational correlation times. Simulations show that OADFA provides important additional information for large molecules (θ ≫ 20 ns), as delayed fluorescence allows us to directly fit the slow-decaying anisotropy. OADFA is directly applied to measuring rotational correlation times of differently sized dye-embedded silica particles and GFP-labeled VLPs in buffer, as well as fluorescent proteins in high viscosity sucrose—all of which exhibit rotational correlation times that are far too long to be measured with ordinary FA. With these approaches, OADFA has ability to measure the molecular volume even when the molecular diameter exceeds 30 nm.

## Supporting Information Available

TEM images of particles, OADFA measurement in high viscosity environments, plasmid and sequence of Qβ coat protein (PDF)
