# Supplementary material for: Sequential Two-Photon Delayed Fluorescence Anisotropy for Macromolecular Size Determination
Source: J Phys Chem B. 2023 Apr 25;127(17):3861–9. doi: 10.1021/acs.jpcb.3c01236 (PMC10165651; doi:10.1021/acs.jpcb.3c01236)
Supplement: Supplementary file 1 — jp3c01236_si_001.pdf [file jp3c01236_si_001.pdf]

Supplementary Information for:

Sequential Two-Photon Delayed Fluorescence Anisotropy for Macromolecular Size Determination

Yi-Han Lu‡, Matthew C. Jenkins‡, Katherine G. Richardson‡, Sayan Palui‡, Md. Shariful Islam‡, Jagnyaseni Tripathy⊥, M.G. Finn‡, Robert M. Dickson‡,*

‡School of Chemistry and Biochemistry and Petit Institute of Bioengineering and Biosciences, Georgia Institute of Technology, Atlanta, GA 30332-0400

⊥ Permanent address: Department of Physics, School of Applied Sciences, KIIT University, Bhubaneswar 751024, India

*Email: dickson@chemistry.gatech.edu

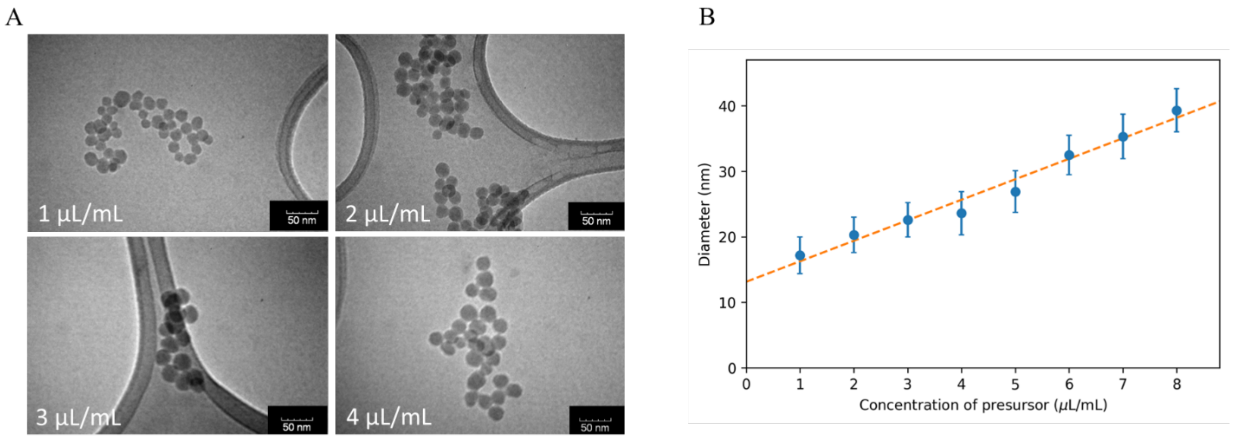

Figure S1. (A) TEM images of rose Bengal silica nanoparticles (RbSNP) synthesized with different concentrations of Rb precursor. Diameter of nanoparticle were measured using the open-source FIJI image analysis software.[1] (B) Concentration of Rb precursor versus RbSNP diameter. The dashed line represents the linear fitting result.

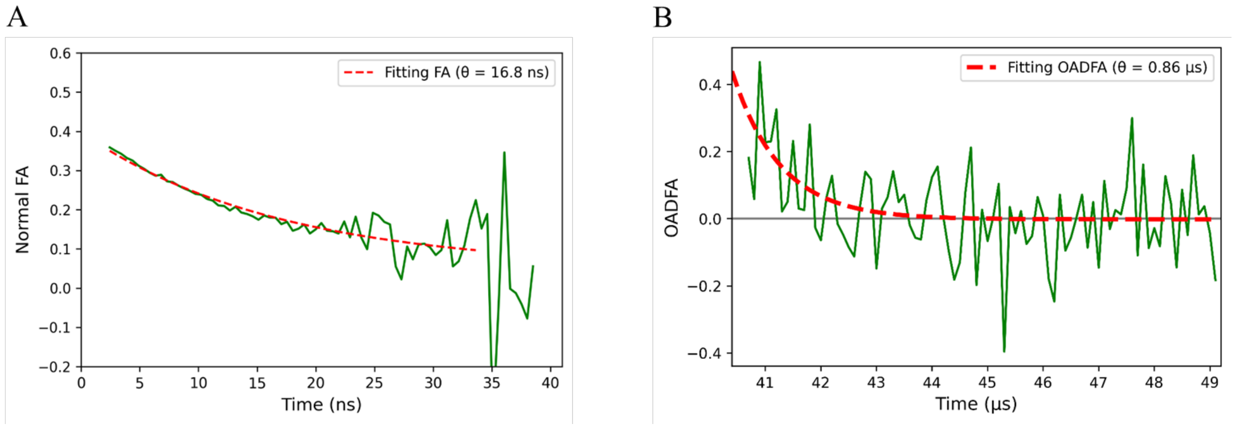

Figure S2. (A) Normal FA result of mVenus in PBS. The fitting result (red dashed curve) gives $r_0 = 0.35$ and $\theta = 16.8$ ns. The fitting only includes the initial part of the curve (time < 35 ns) due to the lost of fluorescence signal at the tail region. (B) OADFA decay of mVenus in the PBS solution with 60% (w/w) sucrose. $\theta = 0.86$ μs from the exponential fitting. The theoretical value of θ under this viscosity (56.76 cP) is 0.81 μs.

A

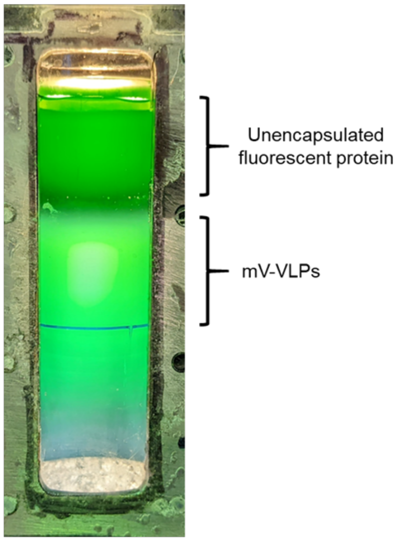

B

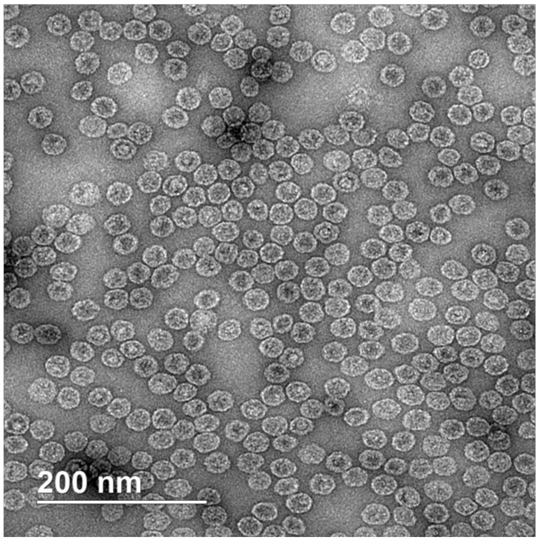

C

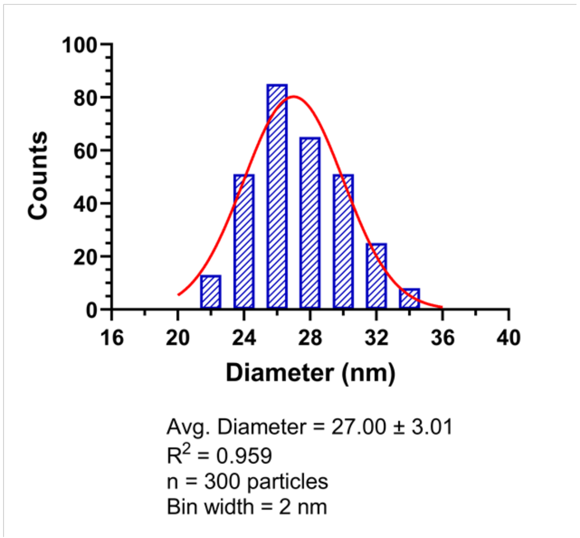

Figure S3. (A) Representative 10-40% sucrose density gradient tube imaged with bottom-up white light illumination. Fluorescent mV-VLPs migrate roughly halfway down the gradient tube during the centrifugation period while unencapsulated mVenus proteins remain nearer to the top of the gradient. (B) TEM image of purified mV-VLPs. (C) Histogram plot of individual VLP diameter measurements from the TEM image in (B) with a Gaussian curve fit. Nanoparticle measurements were collected using the FIJI image analysis software.[1]

pKMJ2 plasmid details:

The pKMJ2 plasmid was generated from the pCDFDuet-1 backbone with the bicistronic segment containing the Qβ coat protein and the Rev-mVenus fluorescent protein gene sequences inserted between the plasmid's NcoI and XhoI restriction sites. Each gene possesses its own ribosomal binding site for independent translation of the two proteins from the same mRNA strand. Packaging of mVenus fluorescent proteins within Qβ VLPs relies on nonspecific electrostatic interactions between the cationic Rev peptide and anionic RNAs that are natively enapsulated within the nanoparticles during *in vivo* expression, as described previously[1-3].

pKMJ2 multiple-cloning site sequence:

TAATACGACTCACTATAGGGGAATTGTGAGCGGATAACAATTCCCCTGTAGAAATAATTTTGTTTAACTTTAAGAAG
GAGATATA**CCATGG**CAAAATTAGAGACTGTTACTTTAGGTAACATCGGGAAAGATGGAAAACAAACTCTGGTCCTCA
ATCCGCGTGGGGTAAATCCCACTAACGGCGTTGCCTCGCTTTCACAAGCGGGTGCAGTTCCTGCGCTGGAGAAGCGT
GTTACCGTTTCGGTATCTCAGCCTTCTCGCAATCGTAAGAACTACAAGGTCCAGGTTAAGATCCAGAACCCGACCGC
TTGCACTGCAAACGGTTCTTGTGACCCATCCGTTACTCGCCAGGCATACGCTGACGTGACCTTTTCGTTCACGCAGT
ATAGTACCGATGAGGAACGAGCTTTTGTTCGTACAGAGCTTGCTGCTCTGCTCGCTAGTCCTCTGCTGATCGATGCT
ATTGATCAGCTGAACCCAGCGTATTAA**GGATCC**CAATTGGGAGCTCGTGTACACGGCGCGCCTGCAGGTCGACAAGC
TTCCCCTGTAGAAATAATTTTGTTTAACTTTAAGAAGGAGATATACTATGGAGCTGACAAGACAGGCACGTCGAAAT
CGCAGGAGACGATGGCGGGAACGTCAAAGGGGCTCGGGTGGCGAGCTCT**CGGCCG**CAGTGAGCAAGGGCGAGGAGCT
GTTCACCGGGGTGGTGCCCATCCTGGTCGAGCTGGACGGCGACGTAAACGGCCACAAGTTCAGCGTGTCCGGCGAGG
GCGAGGGCGATGCCACCTACGGCAAGCTGACCCTGAAGCTCATCTGCACCACCGGCAAGCTGCCCGTGCCCTGGCCC
ACCCTCGTGACCACCCTCGGCTACGGCCTGCAGTGCTTCGCCCGCTACCCCGACCACATGAAGCAGCACGACTTCTT
CAAGTCCGCCATGCCCGAAGGCTACGTCCAGGAGCGCACCATCTTCTTCAAGGACGACGGCAACTACAAGACCCGCG
CCGAGGTGAAGTTCGAGGGCGACACCCTGGTGAACCGCATCGAGCTGAAGGGCATCGACTTCAAGGAGGACGGCAAC
ATCCTGGGGCACAAGCTGGAGTACAACTACAACAGCCACAACGTCTATATCACCGCCGACAAGCAGAAGAACGGCAT
CAAGGCCAACTTCAAGATCCGCCACAACATCGAGGACGGCGGCGTGCAGCTCGCCGACCACTACCAGCAGAACACCC
CCATCGGCGACGGCCCCGTGCTGCTGCCCGACAACCACTACCTGAGCTACCAGTCCAAGCTGAGCAAAGACCCCAAC
GAGAAGCGCGATCACATGGTCCTGCTGGAGTTCGTGACCGCCGCCGGGATCACTCTCGGCATGGACGAGCTGTACAA
GTAA**CTCGAG**TCTGGTAAAGAAACCGCTGCTGCGAAATTTGAACGCCAGCACATGGACTCGTCTACTAGCGCAGCTT
AATTAACCTAGGCTGCTGCCACCGCTGAGCAATAA**CTAGCATAACCCCTTGGGGCCTCTAAACGGGTCTTGAGGGGT**
TTTTTG

Red bold underlined text = Relevant restriction sites

Blue = Ribosomal binding sites

Yellow = Qβ coat protein gene

Red = Rev-peptide

Green = mVenus gene

Bold black text = T7 promoter/terminator

Qβ coat protein:

MAKLETVTLGNIGKDGKQTLVLNPRGVNPTNGVASLSQAGAVPALEKRVTVSVSQPSR
NRKNYKVQVKIQNPTACTANGSCDPSVTRQAYADVTFSFTQYSTDEERAFVRTELAALL
ASPLLIDAIDQLNPAY

Rev-mVenus protein:

MELTRQARRNRRRRWRERQRGSGGELSAAVSKGEELFTGVVPILVELDGDVNGHKFSV
SGEGEGDATYGKLTLKLICTTGKLPVPWPTLVTTLGYGLQCFARYPDHMKQHDFFKSA
MPEGYVQERTIFFKDDGNYKTRAEVKFEGDTLVNRIELKGIDFKEDGNILGHKLEYNYN
SHNVYITADKQKNGIKANFKIRHNIEDGGVQLADHYQQNTPIGDGPVLLPDNHYLSYQS
KLSKDPNEKRDHMVLLEFVTAAGITLGMDELYK

Red = Rev peptide

Green = mVenus

References:

1. Schindelin, J., Arganda-Carreras, I., Fride, E., Kaynig, V., Longair, M., Pietzsch, T., Preibisch, S., Rueden, C., Saalfeld, S., Schmid, B., Tinevez, J.-T., White, D.J., Hartenstein, V., Eliceiri, K., Tomancak, P., Cardona, A. Fiji: an open-source platfrom for biological-image analysis. *Nat. Methods* **2012**, *9*, 676-682.

2. Fiedler, J.D., Brown, S.D., Lau J.L., Finn, M.G. RNA-Directed packaging of enzymes within virus-like particles. *Angew. Chem. Int. Ed.* **2010**, *49*, 9648-9651.

3. Rhee, J.-K., Hovlid, M., Fiedler, J.D., Brown, S.D., Manzenrieder, F., Kitagishi, H., Nycholat, C., Paulson, J.C., Finn, M.G. Colorful virus-like particles: Fluorescent protein packaging by the Qβ capsid. *Biomacromolecules* **2011**, *12*, 3977-3981.

4. Fiedler, J.D., Fishman, M.R., Brown, S.D., Lau, J., Finn, M.G. Multifunctional enzyme packaging and catalysis in the Qβ protein nanoparticle. *Biomacromolecules* **2018**, *19*, 3945-3957.
